# Serosurveillance for *Plasmodium falciparum* Malaria in Peruvian Army Peacekeeping Personnel, Central African Republic, 2021–2022

**DOI:** 10.3201/eid3014.240295

**Published:** 2024-11

**Authors:** Julio A. Ventocilla, Hugo O. Valdivia, Marianela Ore, Rocio Santos, Edson Maguina, Danielle L. Pannebaker, Aida M. Delgado-Flores, Juan F. Sanchez

**Affiliations:** Vysnova Partners, Inc., Alexandria, Virginia, USA (J.A. Ventocilla, R. Santos, E. Maguina); US Naval Medical Research Unit SOUTH, Callao, Peru (H.O. Valdivia, D.L. Pannebaker, J.F. Sanchez); Comando Conjunto del Ejercito del Peru, Lima, Peru (M. Ore); Hospital Militar Central Luis Arias Schrieber, Lima (A.M. Delgado-Flores)

**Keywords:** malaria, vector-borne infections, parasites, *Plasmodium falciparum*, Central African Republic, serologic testing, military personnel

## Abstract

*Plasmodium falciparum* infection threatens military populations deployed to highly malaria-endemic regions, such as Peruvian Army peacekeepers deployed to Central African Republic. During deployment, malaria cases were identified by microscopy and rapid diagnostic tests. After deployment, we performed malaria diagnosis by malachite green loop-mediated isothermal amplification and photo-induced electron transfer PCR assays. We used ELISA to test for *P. falciparum* C-terminal 19-kDa region merozoite surface protein 1–specific IgG from 97 peacekeepers. Malaria prevalence during deployment was 33.33% and we detected 4 cases after deployment: *P. falciparum* (n = 2), *P. ovale* (n = 1), and *Plasmodium* spp. (n = 1). IgG surveillance showed a seroprevalence of 31.96% in peacekeepers, who had a high *P. falciparum* exposure during deployment. Our findings reinforce the necessity of active surveillance in military populations to reduce the risk for introduction of new *Plasmodium* species and strains into the Americas from malaria-endemic areas.

Malaria is a vectorborne disease that affects persons living or traveling within tropical and subtropical regions around the world. According to the World Health Organization, an estimated 249 million malaria cases occurred in 2022 globally; 93.6% of cases and 95.4% of deaths occurred in Africa (*1*). Military personnel are at particularly high risk for malaria during deployments. Malaria caused by *Plasmodium falciparum* can lead to severe symptoms such as fever, chills, headache, and even death, if not identified and treated promptly. Those symptoms can seriously affect the performance of military personnel during the execution of critical operations (*2*). 

A continuous surveillance system using rapid diagnostic tests (RDTs), microscopy, and molecular and serologic diagnostic tools is necessary to determine the absolute risk during deployments to highly malaria-endemic areas. For example, in 2003, a malaria outbreak was reported in 44 US Marines deployed to Liberia who had laboratory-confirmed or suspected *P. falciparum* infections and required immediate medical evacuation (*2*). A review concluded that the outbreak was associated with inefficient preventive measures, such as partial adherence to mefloquine and the inadequate use of repellent and bed nets (*3*). In contrast, military personnel from the United Kingdom successfully deployed to the Democratic Republic of the Congo in the same year and had no reported malaria cases during 512 person-weeks (*4*). Their success was associated with the use of the ABCD (awareness, bite avoidance, chemoprophylaxis, and diagnosis) program to educate and enforce mission objectives (*4*). Both scenarios highlight the role of numerous factors, such as preventive measures, the complexity and objectives of the mission, duration of deployment, and respective risk for malaria transmission (*5*).

The Central African Republic (CAR) also reports high transmission of malaria; ≈2.0 million (36.4%) persons were reported to have suspected or confirmed *P. falciparum* infections during 2021. United Nations military peacekeeping operations in CAR consist of ≈200 armed forces personnel from Peru who promote and maintain the local security of civilians, support democratic efforts, and provide global humanitarian assistance. Peacekeepers from Peru are at risk for severe clinical manifestations of malaria because they are immunologically naive. Infected peacekeepers could introduce new *Plasmodium* species or new strains to the Americas upon their return to Peru from deployment. In this study, we evaluated the exposure to *P. falciparum* malaria infection in military peacekeepers from Peru deployed to CAR during 2021–2022.

## Materials and Methods

### Epidemiologic Information and Blood Collection

**Figure 1:**
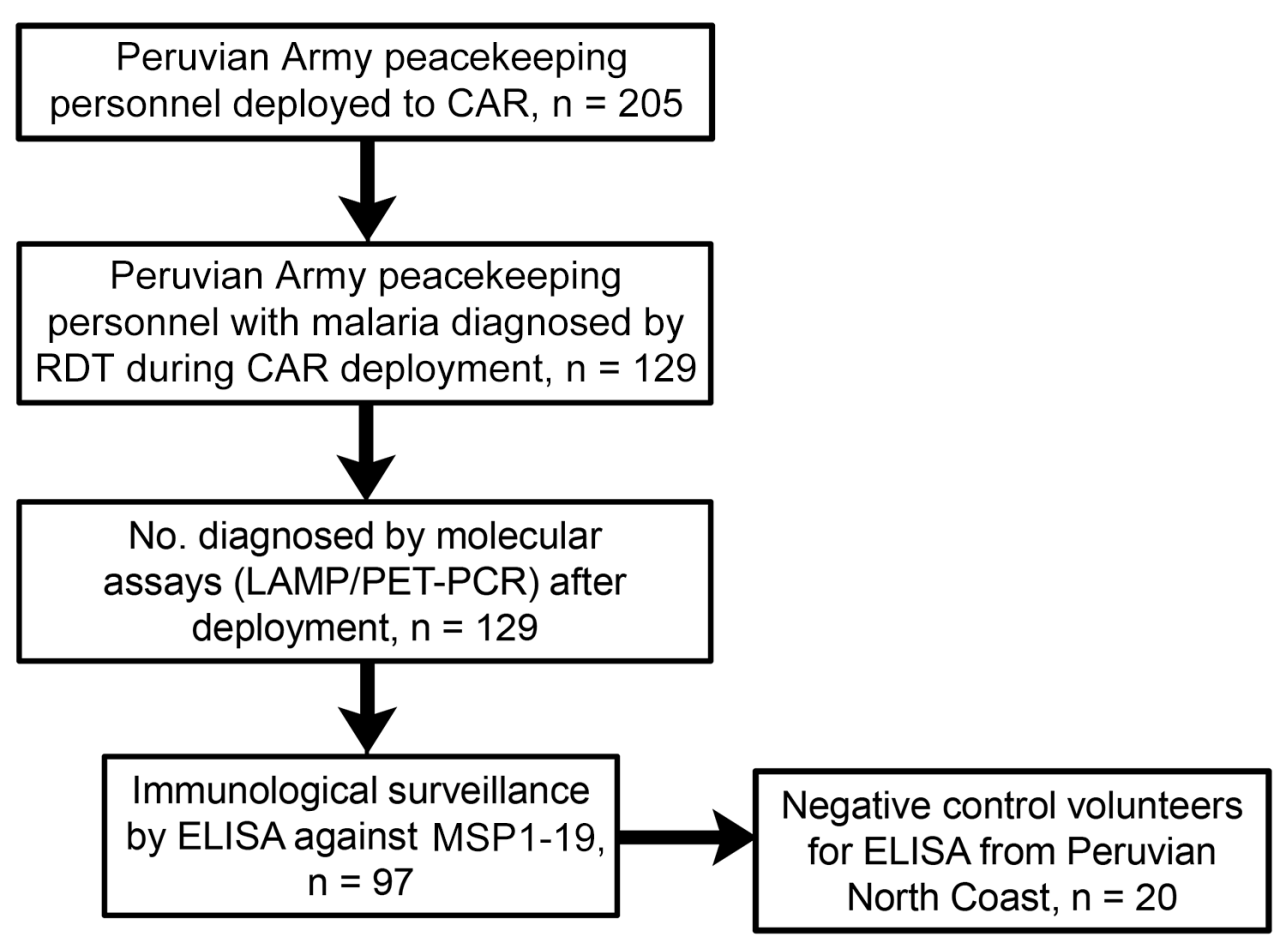
Flow diagram of participants included in study of serosurveillance for *Plasmodium falciparum* malaria in Peruvian Army peacekeeping personnel, Central African Republic, 2021–2022. CAR, Central African Republic; LAMP, loop-mediated isothermal amplification; MSP1-19, C-terminal 19-kDa region merozoite surface protein 1; PET-PCR, photo-induced electron transfer PCR; RDT, rapid diagnostic test.

We collected basic demographic, epidemiologic, and clinical information for malaria case-patients identified in CAR (Figure 1). Whole-blood samples were collected again in Hospital Militar Central Luis Arias Schreiber (the Peruvian Army hospital) in Lima, Peru, 1 month after deployment to evaluate active malaria infection. Upon arrival, military personnel were quarantined for 30 days at an army base in Lima, a nonendemic area for malaria; movement outside the base was completely restricted according to guidelines issued by the Peruvian Army Health Unit. In addition, we randomly selected 97 military peacekeepers (because of limited available testing reagent materials) and collected plasma samples to evaluate exposure to *P. falciparum* (Figure 1).

### Detection of Active Cases

Active *Plasmodium* infection was detected by Boil and Spin malachite green loop-mediated isothermal amplification (LAMP) assay (*6*). In brief, every 20-µL reaction contained 2× in-house LAMP buffer 0.2% Tween-20, 1.5 mol Betaine, 2 mmol of dNTP, 0.004% malachite green dye, 320 U/mL of Bst DNA Polymerase (New England Biolabs, https://www.neb.com), and 5 µL of Boil and Spin DNA template from whole blood. We performed an amplification reaction at 63°C for 60 minutes using a mini heat block (BioExpress, https://www.bioexpress.com). Positive samples showed a green color and were confirmed by 2 independent laboratory technician readers.

We performed malaria species determination using photo-induced electron transfer (PET) PCR (*7*). In brief, the PET genus reaction was performed in a 20 µL volume containing 5 µL of purified DNA from whole blood, 2X TaqMan Environmental Buffer 2.0 (ThermoFisher Scientific, https://www.thermofisher.com), and 250 nmol of genus-forward and FAM-reverse primer. The singleplex PET species-specific reactions contained the same mix but with a concentration of 125 nmol of the HEX-labeled species-specific primer (ThermoFisher Scientific). We used thermal cycling conditions for both genus- and species-specific assays of initial denaturation at 95°C for 10 minutes, 45 cycles of denaturation at 95°C for 10 seconds, and annealing at 60°C for 40 seconds. We used a cycle threshold (Ct) value of <41 to separate positive and negative samples.

### ELISA of Human Samples

We used plasma samples to screen for IgG seropositivity for *P. falciparum* C-terminal 19-kDa region merozoite surface protein 1 (MSP1-19) by indirect ELISA as a marker of *P. falciparum* malaria exposure. We included *P. falciparum*–negative control plasma samples obtained from 20 persons from Piura Department on the north coast of Peru, a region that has very low malaria incidence. We used those plasma samples to calculate a positivity cutoff value using the average optical density value plus 3 SD on the basis of methods published elsewhere (*8*).

### Statistical Analysis

We performed statistical analysis using Stata version 16.1 statistical software (StataCorp LLC, https://www.stata.com) and Prism software version 9 (GraphPad Software, Inc., https://www.graphpad.com). We used descriptive analysis to demonstrate demographic and epidemiologic characteristics of Peruvian Army peacekeepers and used bivariate analysis to compare characteristics between seropositive and seronegative participants.

### Ethics Considerations

Data and sample collections were performed for clinical diagnostic support requested by the Peruvian Army to Naval Medical Research Unit SOUTH. Data and sample analyses were covered by the NAMRU6.2018.0002 protocol (NHSR protocol) approved by Naval Medical Research Unit SOUTH Institutional Review Board. Findings were reported to Peruvian Army authorities for malaria treatment administration, as needed.

## Results

### Study Population

Out of 205 total peacekeepers deployed to CAR during July 2021–June 2022, only 129 (62.9%) were tested for malaria by microscopy or RDT because they reported malaria-like symptoms. Most (92.2%) participants were men, and a high percentage (57.4%) were warrant officers; mean age was 42.4 (SD 7.4) years. The prevalence of malaria during deployment was 33.3% (43/129) by RDT or microscopy, and the number of malaria episodes experienced ranged from 1 to 4. From that group, 79.1% (34/43) had only 1 episode, 16.3% (7/43) had a second episode, and 1 (2.3%) participant reported having third and fourth episodes of malaria-like symptoms (Table 1). All participants received artemether/lumefantrine and responded adequately to malaria treatment.

**Table 1:** Demographic, clinical, and laboratory characteristics of 129 participants in study of serosurveillance for *Plasmodium falciparum* malaria in Peruvian Army peacekeeping personnel, Central African Republic, 2021–2022*

Characteristic	Value
Demographics	
Sex	
M	119 (92.2)
F	10 (7.7)
Mean age, y (SD)	42.4 (7.4)
Rank	
Enlisted	33 (25.6)
Warrant officers	74 (57.4)
Officers	22 (17.0)
Clinical	
Malaria diagnosed in CAR	43 (33.3)
No. malaria episodes, n = 43	
1	34 (79.1)
2	7 (16.3)
3	1 (2.3)
4	1 (2.3)
Malaria diagnosed in Peru	4 (3.1)
Hospitalization required	2 (50.0)
Laboratory, n = 97	
OD values against *P. falciparum* MSP1-19	
Mean (SD)	0.17 (0.31)
Median (IQR)	0.08 (0.07–0.12)
Seroprevalence to *P. falciparum* MSP1-19	
Positive	31 (31.9)
Negative	66 (68.1)

*Values are no. (%) except as indicated. CAR, Central African Republic; IQR, interquartile range; MSP1-19, C-terminal 19-kDa region merozoite surface protein 1; OD, optical density.

In after-deployment samples, LAMP assays detected 4 positive cases out of 129 samples (3.1% positivity), and subsequent molecular method PET-PCR enabled us to further genotype those cases showing 2 *P. falciparum*, 1 *P. ovale*, and 1 *Plasmodium* spp. Of the 4 positive case-patients, 2 (50.0%) reported a malaria episode during their deployment in CAR, and they received the same malaria treatment (artemether/lumefantrine) during deployment as the other positive case-patients.

**Figure 2:**
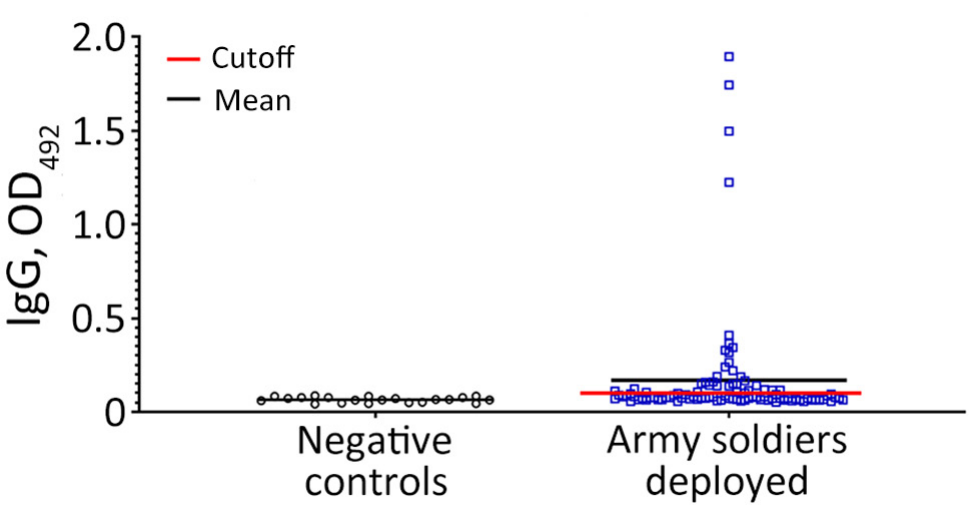
Seroprevalence against *Plasmodium falciparum* malaria in Peruvian Army peacekeepers deployed to Central African Republic, July 2021–June 2022. Dot plot of OD_492_ of *P. falciparum* C-terminal 19-kDa region merozoite surface protein 1 by ELISA assay, with negative control group (black circles, n = 20) and Peruvian Army peacekeepers (blue squares, n = 97), 31 (31.9%) of whom were seropositive. Red line represents the cutoff determined by the average value plus 3 standard deviations of negative OD_492_ control values; black line represents the mean of OD_492_ values per group. OD_492_, optical density at 492 nm.

Regarding malaria exposure, ELISA for MSP1-19–specific IgG demonstrated that 31/97 (31.9%) Peruvian peacekeepers were positive for exposure to *P. falciparum* malaria (Table 1). Four persons had higher IgG titers than the average positive study population, suggesting a recent malaria infection (Figure 2). Finally, seropositivity was statistically significant between participants in whom malaria was diagnosed in CAR and those in whom it was not (p<0.001) and among persons who were enlisted, warrant officers, and officers (p = 0.026) (Table 2).

**Table 2:** Characteristics of 97 participants in a study of serosurveillance for *Plasmodium falciparum* malaria in Peruvian Army peacekeeping personnel, Central African Republic, 2021–2022*

Characteristic	Total, n = 97	Seronegative, n = 66	Seropositive, n = 31	p value
Sex				
M	90 (92.8)	61 (92.4)	29 (93.6)	1.000†
F	7 (7.2)	5 (7.6)	2 (6.4)	
Mean age, y (SD)	43.2 (7.7)	42.7 (7.5)	44.3 (8.0)	0.347
Rank				
Enlisted	24 (24.7)	21 (31.8)	3 (9.7)	0.026
Warrant officers	60 (61.9)	39 (59.1)	21 (67.7)	
Officers	13 (13.4)	6 (9.1)	7 (22.6)	
Malaria diagnosis in CAR				
Y	27 (27.8)	9 (13.6)	18 (58.1)	<0.001
N	70 (72.2)	57 (86.4)	13 (41.9)	

*Values are no. (%) except where indicated. †By 2-tailed Fisher exact test.

## Discussion

Military populations are continuously exposed to *P. falciparum* during deployments in malaria-endemic regions, resulting in outbreaks, mainly in populations with poor preventive measures. However, disease identification on the basis of symptoms alone could lead to an underestimation of actual transmission and subsequently higher risk for malaria during deployments. In our study, we found that almost one third (31.9%) of peacekeepers deployed to CAR were immunologically exposed to *P. falciparum*. We also found a significant difference in seropositivity in military personnel in whom malaria was diagnosed in Africa during deployment. The seronegative participants who were malaria-positive in Africa (13.6%; 9/66) could be explained by antibody kinetics, which require weeks after infection to reach significant levels for detection, especially in naive malaria populations. On the other hand, almost half (41.9%; 13/31) of seropositive participants did not receive a malaria diagnosis; this finding could be caused by exposure to *Plasmodium* parasites that could activate an immune response without a clinical malaria episode. Another possibility could be related to the use of microscopy and RDTs, because these diagnostic methods depend on the skills of laboratory personnel, sample quality, and low parasitemia associated with submicroscopic infections. We do not have information on parasitemia levels to assess the diagnostic capacity of the microscopists and RDTs used during deployment. Diagnosis of active malaria (symptomatic or asymptomatic) should be prioritized using molecular and microscopic tools but could be complemented by serologic analysis in populations with long-term periods of exposure. That finding correlates well with other reports and shows the relevance of serologic surveillance to evaluate exposure to *Plasmodium* parasites in mobile populations like deployed military personnel (*9*). This information highlights the need to improve both preventive measures in military personnel and timing of malaria diagnosis (*2*).

Information is limited about the incidence of malaria in military populations deployed to highly malaria-endemic areas in Africa, especially in social-military conflicted regions. Because of reports of malaria in Somalia and Afghanistan, several military health surveillance systems were implemented (*10*,*11*). The US Defense Medical Surveillance System uses a tracking system to determine the area in which malaria was acquired (*12*). Data from that tracking system are valuable for identifying risk factors related to malaria in military populations, but can be biased by the sample collection process or population type. Identification of military personnel with active malaria infections enables calculation of disease incidence and leads to a better understanding of malaria transmission in this population. However, negative results for malaria on the basis of symptom assessment alone do not necessarily mean that military personnel were not infected during deployment because persons could have been infected without clinical symptoms.

Serologic surveillance in highly malaria-endemic areas offers a tool to determine previous malaria exposure, thereby helping in surveillance efforts to diagnose malaria parasites during symptomatic infection (*13*). Different antigens can be used for diagnosis, including the liver stage antigen-1 and MSP1-19 (*10*,*11*). *P. falciparum* MSP1 antigens, including MSP1-19, are highly immunogenic during blood-stage *Plasmodium* and result in sustained IgG titers up to several months after infection (*14*). Serologic testing can be applied to military populations to determine malaria seroprevalence at the end of deployment, offering complementary information to other diagnostic tools, especially in naive military or civilian populations deployed to highly malaria-endemic regions.

Another relevant finding is the 2 positive non–*P. falciparum* malaria cases diagnosed after deployment. That result differs from the 2023 World Malaria Report, in which CAR reported >2 million malaria cases, 100% of which were *P. falciparum* (*1*). Non–*P. falciparum* malaria has different biologic and clinical manifestations than *P. falciparum* malaria. Accurate *Plasmodium* species diagnosis is key to reducing complications, including relapse because of inadequate malaria treatment for hypnozoites in *P. vivax* or *P. ovale* cases. The 2 cases we detected were not the first reported *P. ovale* cases in military personnel returning to Peru from CAR, highlighting the need for better postdeployment evaluation of personnel to prevent introduction of new *Plasmodium* species (*15*). The possibility of contracting non–*P. falciparum* malaria from other countries in Africa is low because returning personnel were quarantined in Lima, a non–malaria-endemic area, for 30 days after deployment.

The first limitation of our study is that we did not have a predeployment sample to evaluate serologic performance against *P. falciparum* MSP1-19; only self-reports of no previous travel to malaria-endemic regions were available. Second, malaria diagnosis was performed only in symptomatic persons during deployment, and some subjects could have had asymptomatic malaria. Finally, data about compliance to preventive measures (malaria prophylaxis, use of mosquito repellent or bed nets) or other factors that could modify the risk for malaria were unavailable. Those limitations should be considered in future studies in deployed military personnel to highly malaria-endemic areas.

## Conclusions

Our results showed that one third of Peruvian Army military peacekeepers deployed to CAR during 2021–2022 were exposed to *P. falciparum*. Although few sporadic malaria cases were reported in personnel returning from the African region (*15*), those findings reinforce the need for additional tools to measure malaria exposure and to implement preventive measures to reduce malaria risk, thereby decreasing infections in civilian and military populations deployed to highly malaria-endemic areas.
